# Chronic systemic capillary leak syndrome with lymphatic capillaries involvement and MYOF mutation: case report and literature review

**DOI:** 10.3389/fgene.2023.1282711

**Published:** 2023-11-20

**Authors:** Dehua Gao, Wen Zhong, Weiru Zhang, Xuan Wang, Weiping Li, Jun Liu

**Affiliations:** Department of General Medicine, Xiangya Hospital, Central South University, Changsha, China

**Keywords:** chronic systemic capillary leak syndrome, MYOF gene variant, lymphatic capillaries, VEGF, edema, chylous polyserous effusions, hypotrichosis

## Abstract

**Introduction:** Idiopathic systemic capillary leak syndrome (SCLS) is a rare disorder characterized by hemoconcentration, hypoproteinemia and edema. Chronic SCLS (cSCLS) presents as intractable edema, distinguishing it from the classic acute form, and only about 10 cases were reported worldwide. Nevertheless, the underlying pathogenesis of both types is obscure.

**Case presentation:** We report a case of a 58-year-old man with chronic edema persisting for 8 years, complicated by unique chylous polyserous effusions and hypotrichosis, which was successfully relieved by treatment with dexamethasone, intravenous immunoglobulin, and thalidomide. Furthermore, a variant c.5594A>G (p.K1865R) in the *MYOF* gene was identified as a potentially pathogenic mutation through whole-exome genetic sequencing. The proposed mechanism involves its impact on VEGF signaling, leading to increased capillary permeability.

**Conclusion:** Our case illustrates possible lymphatic capillaries involvement in SCLS, which may plays a potential role in immune disorder, and revealed a possible causative genetic mutation of SCLS.

## Introduction

Idiopathic systemic capillary leak syndrome (SCLS), or Clarkson’s disease was first described by Clarkson in 1960 (Clarkson et al., 1960). It is an unusual and life-threatening disease caused by unexplained systemic capillary striking hyperpermeability, and the classic acute SCLS can be described as a triad of “3 Hs”, namely, hypotension, hypoalbuminemia and hemoconcentration (Dhir et al., 2007). However, chronic systemic capillary leak syndrome (cSCLS), a much rarer form of this syndrome, is characterized by persistent, intractable edema rather than fatal episodes of hypovolemic shock. Only 10 cases of cSCLS have been reported since its initial description in 1998 (Table 1) (Rauzy et al., 1998; Stirling et al., 1998; Airaghi et al., 2000; Yderstraede et al., 2005; Keller et al., 2009; Lesterhuis et al., 2009; Datta and Panchamia, 2018; Alkhunaizi et al., 2019; Mullane et al., 2019; Ghurye et al., 2020). SCLS typically affects individuals of Caucasian descent with a median age of onset around 49 years old (Druey and Greipp, 2010; Gousseff et al., 2011). The exact pathological mechanism of cSCLS remains unclear, but it has been observed that approximately 75.4% of SCLS patients have monoclonal immunoglobulinemia (Eo et al., 2018). In addition, several studies have elucidated vascular endothelial growth factor (VEGF) as a potential contributor to endothelial dysfunction in SCLS (Lesterhuis et al., 2009; Kinoshita et al., 2010; Xie et al., 2012; 2014). Besides, VEGF has been reported to play an important role in normal and pathological angiogenesis (Melincovici et al., 2018), and in increasing vascular permeability through the effects of Src family kinases (SFKs) on intercellular junctions and the regulation of focal adhesions (Wautier and Wautier, 2022). Cutaneous histopathology studies revealed inflammatory cell infiltration around vessels, and electron photomicrographs showed endothelial injury in previous literature (Johansson and Löfdahl, 1979; Assaly et al., 2001; Magro et al., 2022). Here, we report a Chinese patient diagnosed with cSCLS, presenting with an 8-year course of chronic edema, along with a rare manifestation of chylous polyserous effusions and hypotrichosis. Moreover, a variant of the *MYOF* gene, which encodes myoferlin, was found in this patient. This variant may be linked to cSCLS via a potential pathological pathway involving VEGF receptor disorder.

**TABLE 1:** A review of literature: clinical characteristics of patients reported with cSCLS.

Author, year and country	Sex/age, y	Presentation	Laboratory findings	Therapy	Response
Rauzy et al., 1998, France [3]	M 34	Anasarca	IgG-κ monoclonal gammopathy (with deposits of IgG on dermal vessel walls)	None	Remission
Stirling et al., 1998, UK [4]	F 30	Anasarca for 8 weeks, pleural effusions	Hypoalbuminemia, IgG-κ > IgG-λ (2 bands) gammopathy,anti-nuclear antibody and rheumatoid factor was positive, and C4 was decreased	Prednisone	Remission
Airaghi et al., 2000, Italy [5]	F 64	Anasarca for 6 months, pleural/pericardial effusions	Hypoalbuminemia, IgG-κ monoclonal gammopathy, bone marrow plasmacytosis 1%	Theophylline, prednisone	Remission
Yderstraede et al., 2005, Denmark [6]	F 51	Anasarca for 3 months, pleural effusion	Hypoalbuminemia, IgG-κ monoclonal gammopathy, peripheral blood and bone marrow examination showed increased T lymphocytes, and flow cytometry revealed a CD4/CD8 ratio of 21/54	Steroids, terbutaline	Remission
Lesterhuis et al., 2009, Netherlands [7]	M 66	Anasarca, pleural effusion	IgA-κ monoclonal gammopathy, elevated transcapillary escape rate of radioactive labeled albumin, elevated VEGF	Bevacizumab	No clinical improvement
Keller et al., 2009, Denmark [8]	M 45	Anasarca for 3 weeks, arthralgias, ascites, night sweats	Hypoalbuminemia, negative monoclonal gammopathy	Prednisolone, azathioprine	Remission
Datta and Panchamia, 2018, USA [9]	F 57	Anasarca for 10 years	Hypoalbuminemia, negative monoclonal gammopathy, elevated VEGF	Terbutaline, montelukast, and prednisone	Unable to tolerate treatment
Mullane et al., 2019, USA [10]	M 54	Anasarca for 2 years, pleural effusion	Hypoalbuminemia, IgG-κ monoclonal gammopathy	IVIG	Remission
Alkhunaizi et al., 2019, Saudi Arabia [11]	F 23	Anasarca for 2 months, pleural effusions and pericardial effusion	Hypoalbuminemia, IgG-κ monoclonal gammopathy	IVIG, theophylline, terbutaline, bevacizumab, intravenous methylene blue, and thalidomide	No clinical improvement
Ghurye et al., 2020, UK [12]	F 52	Anasarca for 1 year, pleural effusions, pericardial effusion	Hypoalbuminemia, IgG-κ monoclonal gammopathy, hypothyroidism	Theophylline, terbutaline, prednisolone, IVIG	Remission
Present case, China	M 58	Anasarca, chylous polyserous effusions, hypotrichosis	Hypoalbuminemia, IgG-κ monoclonal gammopathy, elevated VEGF, destruction of capillary walls and lymphatic capillaries	Dexamethasone, IVIG, and thalidomide	Remission

## Case description

**FIGURE 1:**
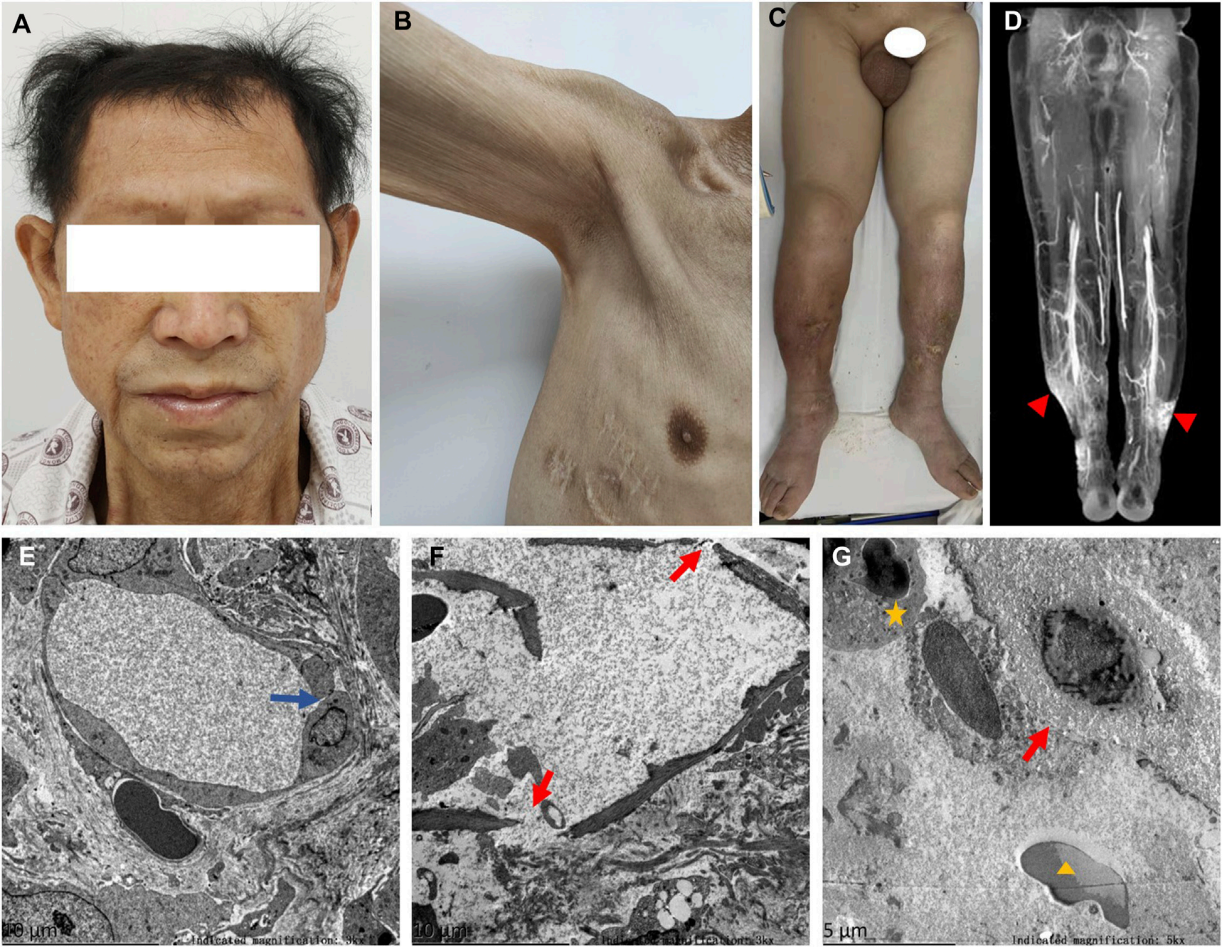
Symptoms, MRL image, and electron photomicrographs of skin biopsy. **(A)** Thinning hair and eyebrows hair. **(B)** Loss of axillary hair. **(C)** Loss of pubic hair and heavy edema of scrotum and both lower limbs. **(D)** Normal lymphatic trunks, but stagnant contrast media at ankle denoting disruption of lymphatic capillaries (▲). **(E)** Disruption of tight junctions between endothelial cells of capillaries (blue arrow). **(F,G)** Fractured capillary walls (red arrows), perivascular neutrophils (★) and erythrocytes (▲).

**FIGURE 2:**
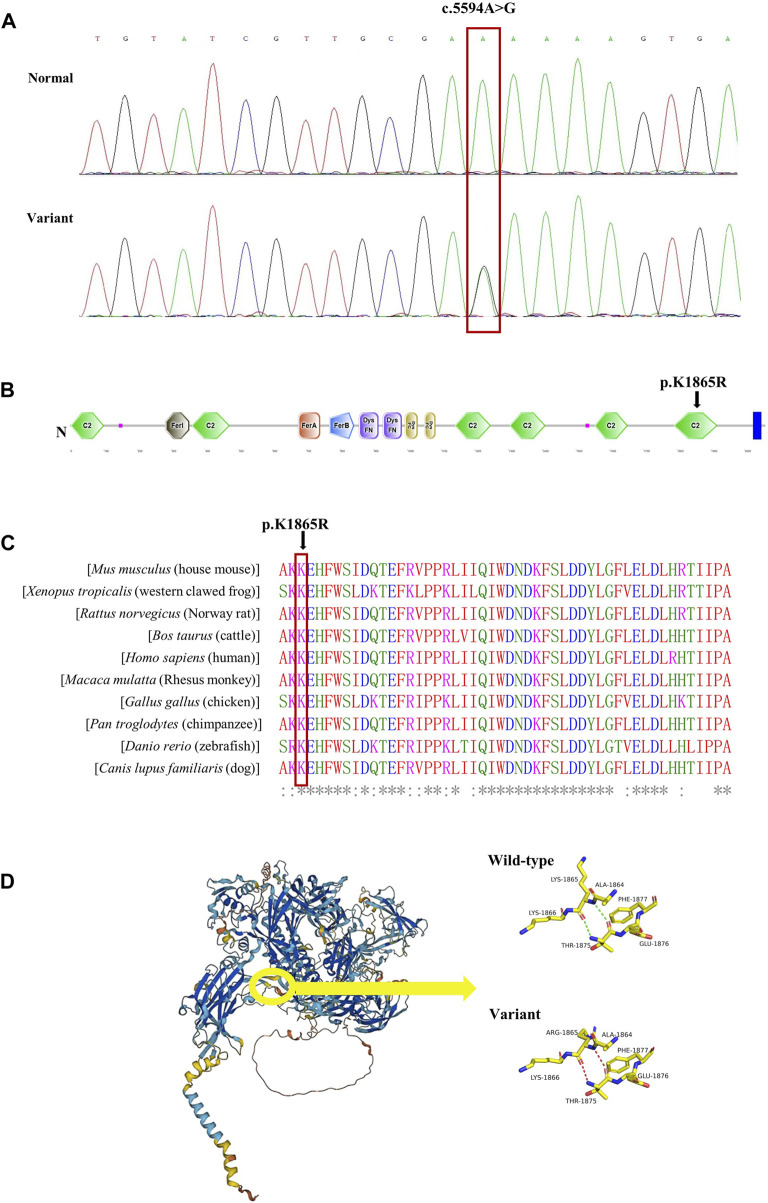
Validation of the missense variant of *MYOF* in the patient. **(A)** Sanger sequencing results from the patient and his son. The heterozygous variant in the *MYOF* gene was identified in the patient (variant), but not in his son (normal). **(B)** The location of the variant in the protein structure of *MYOF*. The arrow indicated the mutated site. **(C)** Amino acid alignment of the *MYOF* protein from several organisms. The position of Lys1865 residue (highlighted by a red box) was highly conserved among different species. **(D)** The 3D structure of *MYOF* protein, and schematic structures of amino acids showed a different steric hindrance of the residue between the WT and the variant.

**FIGURE 3:**
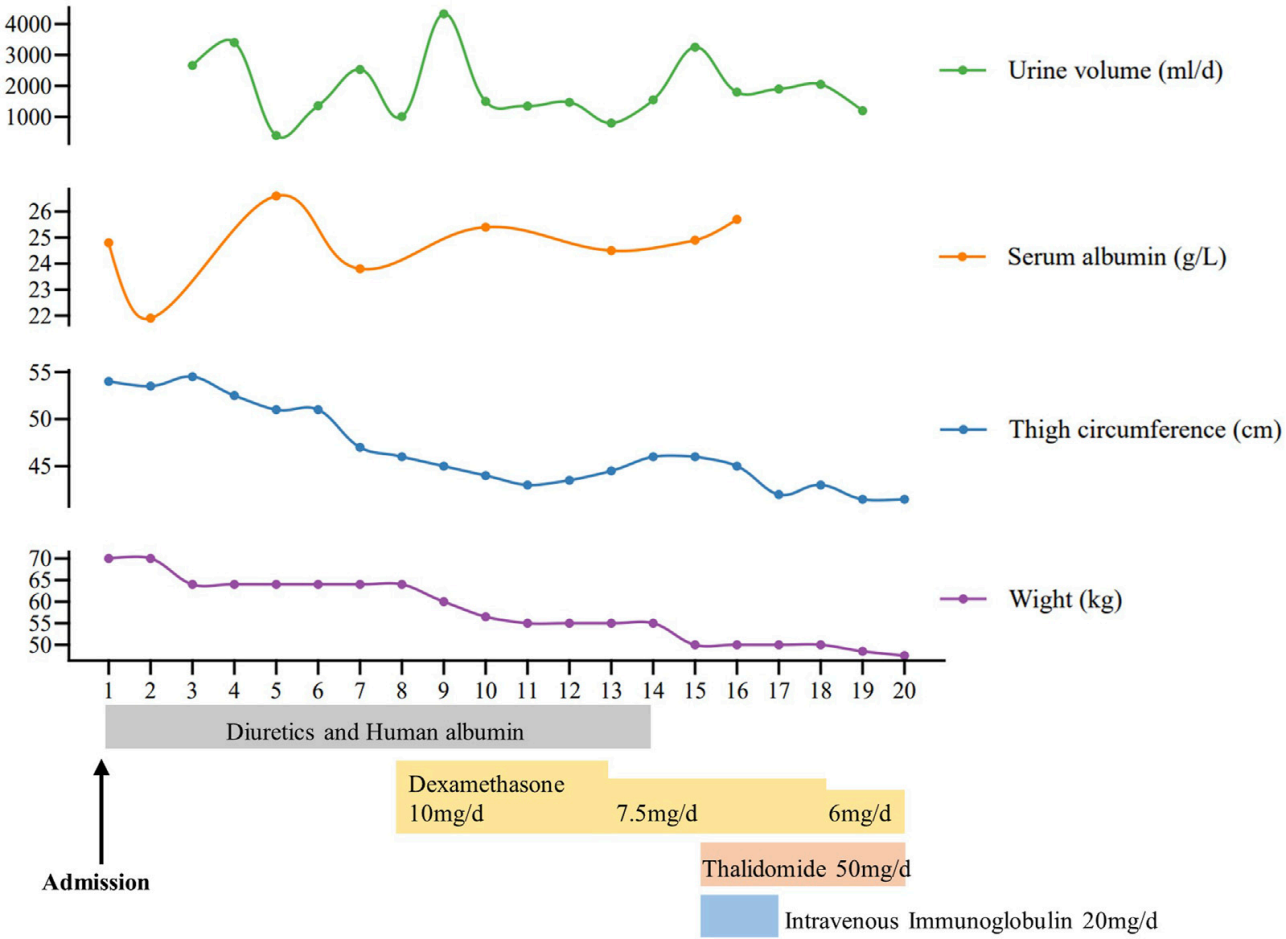
Clinical course of the patient.

A 58-year-old man was referred to our hospital in February 2022 with an 8-year history of systemic edema and loss of eyebrow, axillary and pubic hair (Figures 1A–C). Previous history was unremarkable and no family history of alopecia. The patient’s edema initially presented as symmetrical edema of both lower limbs in 2014, then gradually extended to both upper limbs and the face. Despite receiving treatment with diuretics, the edema continued to progress, with occasional minor relief. Over time, fluid retention escalated, leading to the development of pleural and peritoneal effusions in April 2020. The pleural fluid was found to be chylous, and he had a thoracic duct ligation surgery at a local hospital in January 2021. Shortness of breath and pleural effusion were relieved after the operation, but relapsed 2 months later. No fever, rash or hemodynamic instability was exhibitedduring the course of the disease. After admission, routine laboratory tests were performed. Abnormal blood test results are showed in Supplementary Table S1. The serum albumin measured 24.8 g/L, and the blood IgGκ paraprotein tested weakly positive. We observed mild borderline elevations in white blood cell count, CRP, ESR, IL-1β, and IL-6, while PCT levels remained normal. The complement test revealed a slight decrease in C3 levels, but C4 and the C1 esterase inhibitor levels were within the normal range. Urinalysis, liver, renal and thyroid function tests, as well as cardiac enzymes, NT-proBNP assessments, did not indicate any common diseases associated with edema. Haemoglobin, haematocrit, blood glucose, autoimmune antibodies were all normal. Moderate amount of pericardial effusion and large amounts of bilateral pleural and abdominal fluid were found (Supplementary Figure S1). Thoracentesis and paracentesis were conducted. The hydrothorax and ascites were chyle like, and chylus qualitative test was positive, but no filarial worms were detected. Further, we found out that serum VEGF levels was elevated (264.29 pg/mL, normal values 0–142.2 pg/mL). A bone marrow biopsy revealed 0.1% clonal plasma cells. Additionally, histopathological examination of the skin on the right lower limb exhibited perivascular lymphocytic and plasma cell infiltration, along with suspicious mucinous deposits in the dermis (Supplementary Figure S2). Electron microscopy displayed breakdown of capillary walls (Figures 1E–G). Moreover, magnetic resonance lymphangiography (MRL) of the lower limbs indicated destruction of lymphatic capillaries, although the lymphatic trunk appeared normal (Figure 1D). Meanwhile, he and his son underwent whole-exome sequencing and Sanger sequencing, respectively, and the patient’s genomic DNA sample contained the potentially causal heterozygous mutation (c.5594A>G (p.K1865R)) in the *MYOF* gene, while his son’s did not (Figure 2A). In light of these findings, a multidisciplinary team diagnosed cSCLS as the primary condition. Subsequently, a treatment regimen was initiated, with the patient prescribed 10 mg of dexamethasone per day for 4 days, followed by a reduction to 5 mg for maintenance. Additionally, 20 mg of intravenous immunoglobulin (IVIG) was administered. Thalidomide was then introduced at a daily dose of 50mg, and human albumin infusion was conducted intermittently. Fortunately, he was discharged from the hospital after experiencing a remarkable 22.5 kg weight loss and significant relief from overall edema symptoms. The clinical course of the patient is shown in Figure 3. Presently, he has ceased all medications and maintains relatively good health, with only mild limb edema remaining.

We conducted bioinformatics analysis of the *MYOF-1865R* variant in our patient and suspect it was pathogenic. The *MYOF-1865R* variant was very rare, which was identified only 0.000472,233, 0.0008702 and 0.00006052 in Chinese, East Asian and global populations from the ChinaMap database and GnomAD. Furthermore, the variant was not observed in non-East Asian populations. The missense variation resulted in arginine replacing lysine in amino acid sequence 1865, which was in the conserved C2F domain of *MYOF* in humans and other species. (Figures 2B,C). Furthermore, we employed web-based software tools to assess the pathogenicity of the genetic variant, specifically, Mutation Taster (0.999, disease causing), CADD (22.3, potentially deleterious) and REVEL (0.338, neutral). The 3D structure of *MYOF* protein was created using the AlphaFold2. The mutant protein showed a different steric hindrance of the residue (the new residue has a smaller size), which may lead to protein misfolding, resulting in pathogenicity (Figure 2D).

## Discussion

The patient experienced 8 years of persistent systemic edema and refractory hypoalbuminemia, along with unusual hair loss throughout the body and chylous polyserous effusions. Electron microscopy and MRL examination revealed the destruction of capillary walls and lymphatic capillaries (LC), respectively. After ruling out common diseases that could cause edema, the patient was ultimately diagnosed with cSCLS. Treatment with dexamethasone, IVIG, and thalidomide brought relief from the edema. Additionally, we identified a missense variant of the *MYOF* gene in this patient, suggesting a potential association with the development of cSCLS.

Differing from the traditional triad of SCLS, which typically includes rapid onset of hypotension (systolic blood pressure <90 mmHg), hemoconcentration (hematocrit >49%–50% in men and 43%–45% in women), and hypoalbuminemia (<3.0 g/dL) (Druey and Parikh, 2017), cSCLS is characterized by chronic systemic edema and hypoalbuminemia, and possibly serous cavity effusion, without episodes of hemodynamic instability (Kapoor et al., 2010). However, there is currently no consensus on diagnostic criteria or specific biomarkers for cSCLS, and other diseases need to be ruled out before a final diagnosis can be made. In our patient, the main clinical manifestations were chronic edema and hypoalbuminemia, and common disorders associated with edema, such as cardiac, renal, and hepatic insufficiency, as well as hypothyroidism, were ruled out through routine examination. But our patient exhibited unusual chylous effusion and hair loss, which had not been reported in previous cases of SCLS (Eo et al., 2018). The presence of chylous effusion suggested the possibility of a lymphatic duct leak, warranting consideration. Furthermore, the patient experienced body hair loss, which, from a dermatological perspective, could be linked to an immune disorder (Zhou et al., 2021). However, capillary walls disruption observed under electron microscopy suggested endothelial cell (ECs) dysfunction, which was in line with the characteristics of SCLS (Johansson and Löfdahl, 1979; Assaly et al., 2001; Druey and Greipp, 2010; Baloch et al., 2018). The skin biopsy seemed toreveal several diagnostic clues (Magro et al., 2022), showing perivascular lymphocytic and plasma cell infiltration, consistent with previous reports (Cicardi et al., 1997; Fardet et al., 2004; Magro et al., 2022). Besides, elevated levels of VEGF and the presence of monoclonal gammopathy of unknown significance (MGUS) served as supportive diagnostic evidences for SCLS (Druey and Parikh, 2017; E, M and J, 2017). The remarkable effectiveness of the treatment also in turn validated our diagnosis of cSCLS.

The immune-mediated hypothesis regarding the pathogenesis of SCLS has been extensively discussed in prior studies (Cicardi et al., 1997; Dowden et al., 2009; Baloch et al., 2018; Wu et al., 2021). Evidence of immune involvement includes perivascular lymphocytic infiltration in the skin (Cicardi et al., 1997; Fardet et al., 2004; Magro et al., 2022) and the observation of elevated peripheral blood T cells (Cicardi et al., 1990; Dowden et al., 2009). Notably, it has been reported that patients with higher levels of monoclonal components are more prone to severe recurrences (Pineton de Chambrun et al., 2017), underscoring the significance of immune dysregulation in the pathophysiology of SCLS. Furthermore, various drugs targeting immune system dysfunction have shown effectiveness in treating the disease, including IVIG, corticosteroids, infliximab, thalidomide, and others. Among these empirically employed treatments, prophylactic IVIG stands out as the most effective approach for reducing mortality, owing to its numerous immunomodulatory properties (Eo et al., 2018).

It has been widely reported that VEGF levels are elevated in many SCLS patients, coinciding with the course of the disease (Lesterhuis et al., 2009; Kinoshita et al., 2010; Xie et al., 2014). Bevacizumab has been suggested as a potential treatment for SCLS patients (Kouadri et al., 2021), and VEGF may be responsible for the immune systems disorder (Xie et al., 2012; Mo et al., 2020). VEGF/VEGFR-2 is an important angiogenic signaling pathway that also plays a potential role in lymphangiogenesis (Liu and Yu, 2022). The lymphatic system is crucial for immune regulation, and its role in the development of various diseases was gradually being revealed, especially lymphatic capillaries was highlighted by scientists for further study (Liu and Yu, 2022). Considering that high VEGF levels, lymphatic capillaries destruction, and chylous effusion were observed in our patient, we hypothesized that high VEGF levels in SCLS patients would lead to lymphatic capillaries damage and chylous polyserous effusions discovered in our patient, and possibly further connect with immune disorder in SCLS. In addition, we identified a variant of the *MYOF* gene, which encodes Myoferlin, a type II transmembrane protein highly expressed in ECs (Lek et al., 2012). It functions in VEGF signaling by regulating VEGFR-2 stability, thereby affecting VEGF-induced vascular permeability (Bernatchez et al., 2007). It is worth noting that the mutation of *MYOF* gene (c.651G>T, p. R217S) have also been reported in hereditary angioedema (HAE), a disease characterized by vascular leakage. And the *MYOF-217S* variant in HAE increased the VEGFR-2 localization on the cell membrane, which could raise VEGF-C levels (Ariano et al., 2020). Furthermore, some related genes like *EDNRA*, *ARHGAP5*, and *MYOF* were discussed in SCLS (Xie et al., 2013; Sek et al., 2015; Pierce et al., 2017). And the variant of *MYOF* in SCLS is one of the top-ranked single nucleotide polymorphisms (SNPs) detected by prior researchers (Xie et al., 2013). Therefore, it was assumed that MYOF-VEGF signal pathway disorder might induce the endothelial dysfunction in SCLS which needs further investigation.

## Conclusion

We report a case of cSCLS characterized by persistent edema, with unusual chylous effusion and hypotrichosis. The treatment of dexamethasone, IVIG, and thalidomide was significantly effective in relieving edema. Additionally, a *MYOF* gene mutation (c.5594A>G, p. K1865R) was found in our patient, which might be a suspected pathological gene for SCLS.

## Data Availability

The datasets for this article are not publicly available due to concerns regarding participant/patient anonymity. Requests to access the datasets should be directed to the corresponding author.
