# Corrigendum: Chronic systemic capillary leak syndrome with lymphatic capillaries involvement and MYOF mutation: case report and literature review

**DOI:** 10.3389/fgene.2024.1506453

**Published:** 2024-10-21

**Authors:** Dehua Gao, Wen Zhong, Weiru Zhang, Xuan Wang, Weiping Li, Jun Liu

**Affiliations:** Department of General Medicine, Xiangya Hospital, Central South University, Changsha, China

**Keywords:** chronic systemic capillary leak syndrome, MYOF gene variant, lymphatic capillaries, VEGF, edema, chylous polyserous effusions, hypotrichosis

In the published article, there is an error in the **Funding** statement. The correct grant number for the Science and Health Collaboration Fund Project of Natural Science Foundation of Hunan province is 2022JJ70159. The correct **Funding** statement appears below:

