# Supplementary material for: Chronic systemic capillary leak syndrome with lymphatic capillaries involvement and MYOF mutation: case report and literature review
Source: Front Genet. 2023 Nov 20;14:1282711. doi: 10.3389/fgene.2023.1282711 (PMC10694220; doi:10.3389/fgene.2023.1282711)
Supplement: Supplementary file 1 [file Table1.DOCX]

| **Table S1 Abnormal blood test results of the patient.** |  |
| --- | --- |
| **Laboratory test** | **Measurement results** |
| Complete blood count |  |
| White blood cell count (3.5-9.5 × 10^9^/L) | 11.2 ↑ |
| Neutrophil count (2-7×10^9^/L) | 9.3 ↑ |
| Hemoglobin (130-175 g/L) | 127 ↓ |
| Platelets (125-350 × 10^9^/L) | 478 ↑ |
| Biochemistry |  |
| Total protein (65-85 g/L) | 49.5 ↓ |
| Albumin (40-55 g/L) | 24.8 ↓ |
| C-reactive protein (0-8 mg/L) | 14.7 ↑ |
| Erythrocyte sedimentation rate (0-21 mm/h) | 29 ↑ |
| interleukin-1β (<5 pg/ml) | 5.38 ↑ |
| interleukin-6 (<5.9 pg/ml) | 8.94 ↑ |
| Coagulation function |  |
| Fibrinogen (2-4 g/L) | 4.46 ↑ |
| Fibrin degradation products (0-5 mg/L) | 10.1 ↑ |
| D-dimer (0-0.5 mg/L) | 0.97 ↑ |
| Sex hormone panel |  |
| estradiol (94.8-223 pmol/L) | 65.65 ↓ |
| testosterone (6.68-25.7 nmol/L) | 2.89 ↓ |
| Immunological tests |  |
| IgGκ paraprotein (negative) | weakly positive |
| Complement C3 (790-1520 mg/L) | 772 ↓ |
| IgG (7-16 g / L) | 6.84 ↓ |
| Vascular endothelial growth factor (0-142.2 pg/mL) | 264.29 ↑ |
