# Supplementary figures and images for: Chronic systemic capillary leak syndrome with lymphatic capillaries involvement and MYOF mutation: case report and literature review
Source: Front Genet. 2023 Nov 20;14:1282711. doi: 10.3389/fgene.2023.1282711 (PMC10694220; doi:10.3389/fgene.2023.1282711)

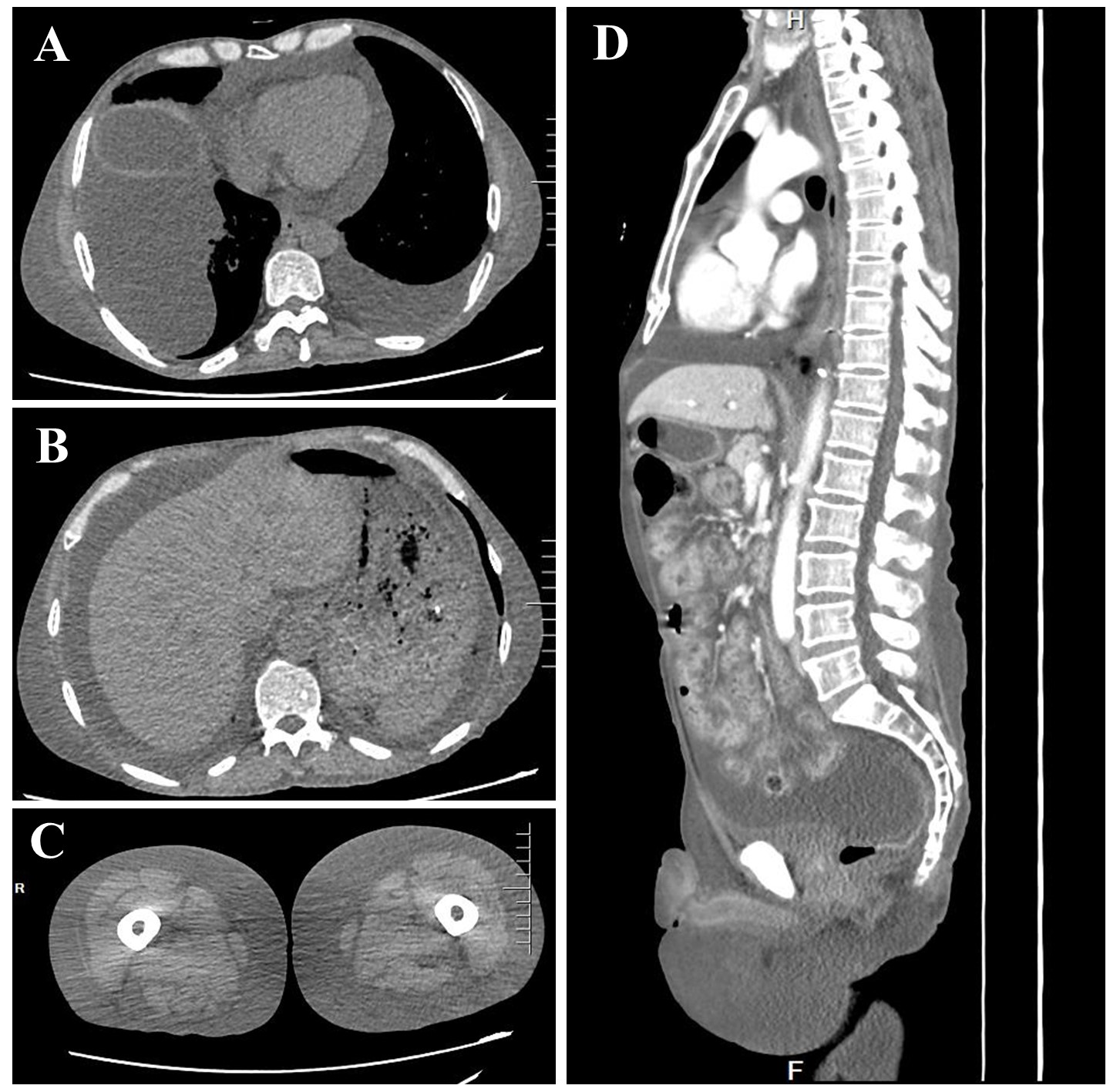

Supplement: Supplementary file 2 [file Image1.JPEG]

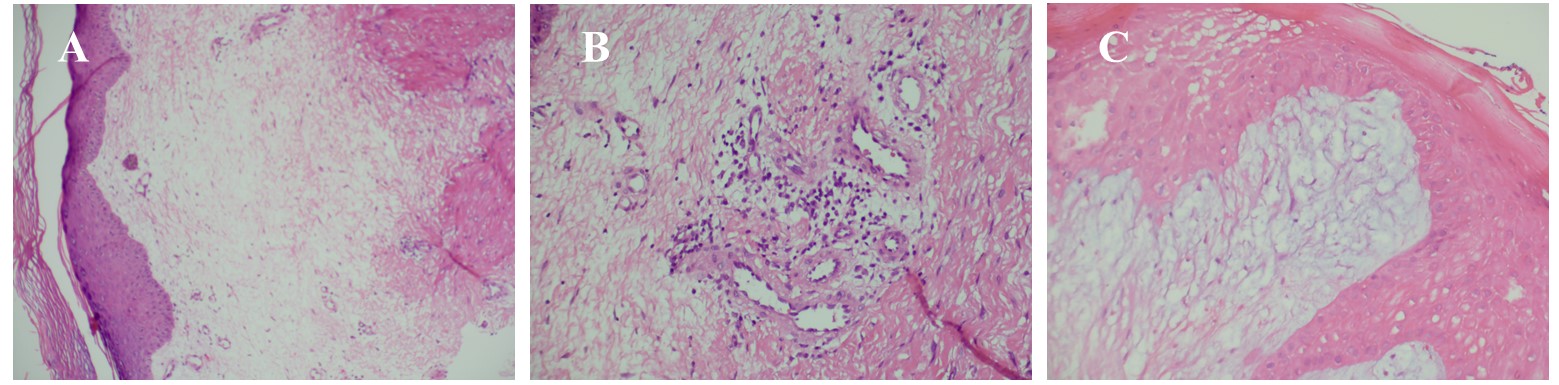

Supplement: Supplementary file 3 [file Image2.JPEG]
